# Asymptomatic Meckel’s diverticulum in adults: Is diverticulectomy indicated?

**DOI:** 10.4103/1319-3767.70626

**Published:** 2010-10

**Authors:** Vipul D. Yagnik, Bhargav D. Yagnik

**Affiliations:** Ronak Endo-laparoscopy and General Surgical Hospital, Patan, India. E-mail: vipul.yagnik@gmail.com; 1 General Practitioner, Ahmedabad, Gujarat, India

Sir,

We read the article entitled” Asymptomatic Meckel’s diverticulum in adults: Is diverticulectomy indicated?”[1] with interest. We would like to congratulate the authors for conducting a study on such a large number of patients of acute abdomen. The current trend is toward prophylactic diverticulectomy because of the availability of laparoscopy for the management of Meckel’s diverticulum. Optimal management of incidentally found Meckel’s diverticulum is controversial, even though resection is associated with a low risk of complications.[2] One recent small study recommended prophylactic diverticulectomy in less than 50-years-old individuals. According to one large series, the possibility of a Meckel’s diverticulum to become symptomatic in an adult patient is less than 2%, while morbidity rates of incidentally removed Meckel’s diverticula were as high as 12% in few studies and 400 asymptomatic diverticula would have to be resected to save one patient.[3] Out of 8 asymptomatic patients in this study,[1] 6 patients (75%) were males and 7 were young < 45 years (87.5%). Though authors have not mentioned about the presence of band and length of diverticulum, from the information available, at least five patients had a risk score[4] of at least 6 and were candidates for resection. We do not agree with the strong recommendation of prophylactic diverticulectomy for all patients as indicated in the present study[1] as sample size is too small (8 patients) to recommend prophylactic diverticulectomy and 12.5% morbidity[1] in such a small study is too high. We believe there is a need for well designed randomized controlled trials to justify prophylactic removal of Meckel’s diverticulum, and until the results become available, it would be a better option to remove the diverticulum on the basis of the risk score.[4]
